# Diagnostic accuracy of rapid and point-of-care tests for *Schistosoma haematobium* in African populations: a systematic review and meta-analysis

**DOI:** 10.3389/fpara.2026.1830789

**Published:** 2026-07-22

**Authors:** Erica Draxl, Eleanor Ochodo, Pavitra Pillay, Ntombizodwa Ndlovu

**Affiliations:** 1 School of Public Health, Faculty of Health Sciences, University of Witwatersrand, Johannesburg, South Africa; 2 Centre for Global Health Research, Kenya Medical Research Institute, Kisumu, Kenya; 3 Centre for Evidence-Based Health Care, Department of Global Health, Faculty of Medicine and Health Sciences, Stellenbosch University, Stellenbosch, South Africa; 4 Department of Biomedical and Clinical Technology, Faculty of Health Sciences, Durban University of Technology, Durban, South Africa

**Keywords:** field-based diagnostic tests, low-resource settings, rapid diagnostic tests, Schistosoma haematobium, urogenital schistosomiasis

## Abstract

**Background:**

Accurate and accessible point-of-care testing for urogenital schistosomiasis (UGS) is vital for control and prevention in sub-Saharan Africa. Point-of-care (POC) rapid diagnostic tests with high sensitivity for both high and low parasite burdens have been developed. These tests need to be assessed for their applicability in poorly resourced UGS-endemic areas so that control programs can be initiated, and improved screening and routine diagnosis can be implemented.

**Methods:**

This diagnostic test accuracy (DTA) review evaluates the sensitivity and specificity of eligible field-based index tests compared to laboratory-based reference tests for the diagnosis of UGS among individuals from Africa.

Four databases were systematically searched for DTA studies on *S. haematobium*-exposed patients in endemic settings from 2000 to 2026. Methodological quality was assessed using the QUADAS-2 tool. Using the variability of test thresholds, a bivariate or HSROC model was used to calculate the diagnostic performance for each eligible test. Heterogeneity and subgroup analyses were conducted for the following variables: reference-standard comparators, age groups, and prevalence settings.

**Results:**

Twenty-nine studies were evaluated, in which nine different POC and rapid tests were eligible, and four were included in a meta-analysis. Two field-based molecular tests, loop-mediated isothermal amplification (LAMP) and recombinase polymerase amplification (RPA), demonstrated high accuracy (sensitivity, 94.7%; specificity, 66.4%; and sensitivity, 94.1%; specificity, 96.6%, respectively). The immunochromatographic-rapid diagnostic test (ICT-RDT) and *S. mansoni* cercarial transformation fluid RDT (SmCTF-RDT) serological tests are easy-to-use POC cassettes and have high sensitivity (98.6% and 98.4%, respectively) but require a portable centrifuge to obtain serum samples. Other serological tests: dipstick dye immunoassay (DDIA) and indirect haemagglutination assay (IHA) had lower sensitivities (64.8% and 59.5%, respectively). The POC circulating cathodic antigen test (POC-CCA) demonstrated a sensitivity of 49.8% and specificity of 77.0% and has low diagnostic accuracy for *S. haematobium*. Portable and mobile-phone microscopes (sensitivity, 81.5%, specificity, 91.4%) were less sensitive than conventional microscopy but can have benefits in field-based low-resource laboratories.

**Conclusion:**

Further validation with improved reference standards is required to assess the diagnostic accuracy of many of the tests identified. RPA and LAMP emerged as promising field-applicable rapid tests for schistosomiasis control.

## Introduction

Schistosomiasis, or bilharzia, is a water-borne neglected tropical disease caused by the parasitic blood flukes of the genus *Schistosoma*. Schistosomiasis affects more than 150 million people worldwide, 84.3% of whom live in sub-Saharan Africa (SSA) (Li et al., 2025). Two types of *Schistosoma* species exist in SSA: *Schistosoma haematobium*, which causes urogenital schistosomiasis, and *Schistosoma mansoni*, which causes intestinal schistosomiasis (Gray et al., 2011). Both are acquired by exposure to infected water harboring specific snail species that serve as intermediate hosts for *Schistosoma* species (Adenowo et al., 2015). Intestinal schistosomiasis results in lesions in the intestinal mucosa and liver, which can result in bleeding, malnutrition, advanced disease manifestations, and even death due to fibrosis in the portal veins of the liver. Urogenital schistosomiasis results in pathology causing damage to the bladder, kidneys, and genital organs, leading to symptoms such as painful urination and hematuria, and can advance to bladder cancer or obstructive uropathy (Gray et al., 2011).

Chronic or long-standing schistosomiasis results in lifelong debility due to malnutrition, internal hemorrhage, and end-stage organ failure, contributing to 1.746 million disability adjusted life years (DALY’s) lost annually (Shen and Luo, 2025). *S. haematobium* infection in the genital tract is called female or male genital schistosomiasis (FGS or MGS, respectively) and results in an increased risk of acquiring sexually transmitted diseases, including HIV (Sturt et al., 2020). Numerous studies in SSA have ascertained that FGS is associated with a 2.9 to 4-times higher risk of HIV acquisition (Sturt et al., 2020).

Current schistosomiasis control and elimination strategies rely on periodic mass drug administration (MDA) with a global target of regular treatment of at least 75% of school-aged children at risk (World Health Organization, 2022). Effective and efficient MDA requires adequate surveillance (Stothard et al., 2009), particularly for schistosomiasis-endemic regions. However, only half of the target populations in endemic SSA countries currently receive MDA (Hong, 2023), and many countries are unable to initiate control programs due to challenges in implementation (Randjelovic et al., 2015). Increasingly, the need for improved diagnostic tools has been recognized by the World Health Organization (WHO) and schistosomiasis research communities as an imperative first step to improve control strategies, and for improved geographic mapping and prevalence in endemic areas (Hong, 2023). Fast, reliable, and near-patient diagnostic tests are an integral part of sustained control programs and decision-making for schistosomiasis elimination programs.

There is a notable gap in knowledge regarding diagnostic tests designed to detect *S. haematobium* in low-resource settings, where access to laboratory equipment is limited. Although microscopy of filtered urine for egg detection is considered the reference or “gold standard” diagnostic method, it has significant limitations (Hong, 2023). The sensitivity of microscopy is reliable primarily where high infection intensity occurs endemically or in ‘hotspots’ where infection rates exceed 50% (World Health Organization, 2022). However, in low-prevalence regions and among individuals with chronic infections, its sensitivity is markedly reduced, hindering early diagnosis and timely treatment (Hong, 2023). For instance, in women with chronic schistosomiasis diagnosed with female genital schistosomiasis (FGS), only 23% were found to have detectable eggs in urine samples using microscopy (Poggensee et al., 1998). This discrepancy highlights the urgent need for more sensitive diagnostic tools to replace conventional parasitological methods, particularly for accurate prevalence assessments and improved case detection.

The ideal test for resource-limited settings would be a direct point-of-care (POC) test conducted at or near the clinical site that is rapid, easy to use, and highly sensitive and specific for all *Schistosoma* species. An appropriate rapid turnaround time between performing the test and obtaining the result is 10 minutes to 2 hours (World Health Organization, 2014). Such a test would support timely decision-making and improve patient management (Drain et al., 2014).

Direct diagnostic methods include antigen tests and molecular (DNA-based tests), all of which identify the schistosome parasite itself and are therefore considered more accurate for prevalence estimation, surveillance, and case detection (Chala, 2023). In contrast, indirect tests do not detect the parasite directly. These include urine reagent strips and antibody-based serological tests, which serve as proxy measures of infection and are often used for screening. Although serological tests are indirect, they remain clinically valuable because they detect antibodies, which are the host’s immune response to *Schistosoma* infection. Anti-*Schistosoma* antibodies may persist for life in untreated infections. For this reason, serology can support the diagnosis of asymptomatic or untreated chronic schistosomiasis, particularly when eggs or other antigens are difficult to detect by other methods. After treatment with praziquantel, anti-*Schistosoma* antibodies may take approximately 72–100 weeks to become undetectable (González-Sanz et al., 2025).

Serological tests are therefore useful as rapid, low-cost screening tools in individuals with untreated chronic infections, as well as selected newly infected populations, including recent travellers or migrants returning from endemic areas (González-Sanz et al., 2025) and young children with new infections (Wami et al., 2014).

For real field applications demanded by resource-limited settings, the ASSURED criteria (Affordable, Sensitive, Specific, User-friendly, Robust & Rapid, Equipment-free, Deliverable) for point-of-care diagnostic tests were developed by the WHO in 2003 (Mabey et al., 2004), and updated to REASSURED in 2019, where Real-time connectivity and Ease of specimen collection were included in the diagnostic framework (Land et al., 2019). Currently, the commercially available easy-to-use lateral flow POC-CCA test is considered accurate for *S. mansoni* and meets ASSURED criteria (Danso-Appiah et al., 2016), however its accuracy is low for *S. haematobium*, making it inaccurate for areas of co-endemicity (Ochodo et al., 2015; Rubaba et al., 2018).

Despite the need, the optimal approach for rapid diagnosis of urogenital schistosomiasis has not been fully established. A recent diagnostic test accuracy (DTA) review in the literature assessing tests for both *S. haematobium* and *S. mansoni* found no viable cost-effective practical alternatives to conventional microscopy for *S. haematobium-*prevalent areas (Vaillant et al., 2024). There is a need to synthesize the available evidence on POC and rapid tests for the rapid diagnosis of *S. haematobium*, particularly in low-resource settings with varying levels of disease prevalence and chronicity. Recent advancements in the development of low-cost, field-based versions of existing expensive laboratory-based tests also warrant attention, as they hold promise for enhancing surveillance and control efforts in endemic and non-endemic areas (Ally et al., 2024).

In this systematic review, we assessed and compared the diagnostic accuracy of eligible rapid and point-of-care tests with a timing of approximately 2 hours for the detection of *S. haematobium* infection, using appropriate laboratory tests as the reference standard in exposed African populations. We also compared the diagnostic accuracy of eligible tests in different areas of prevalence (endemic high/moderate/low), age-groups (children and adults), and for different reference tests (conventional microscopy, PCR, composite tests) using subgroup analysis. This review aims to identify appropriate POC or rapid tests that can serve as a suitable replacement for conventional microscopy in the formulation of integrated control strategies in low-resource settings.

## Methods

This systematic review of diagnostic test accuracy (DTA) followed the Preferred Reporting Items for Systematic Reviews and Meta-Analyses-Diagnostic Test Accuracy (PRISMA-DTA) guidelines (Mcinnes et al., 2018) and the Cochrane DTA handbook (Dinnes et al., 2022).

### Search strategy and study selection

Four major databases, PubMed, Global Health, ProQuest, and the Web of Science, were systematically searched for eligible DTA studies published between January 2000 and June 2026. The medical subject headings (MeSH) search terms used were (*Schistosoma haematobium*) AND (diagnostic test) AND (point of care test) AND (diagnostic accuracy) OR (Urogenital schistosomiasis) AND (diagnostic test) OR (*Schistosoma haematobium*) AND (rapid diagnostic test) OR (Diagnostic accuracy) AND (*Schistosoma haematobium*). A second search strategy and article mining were conducted for individual index tests after the initial search to ensure that the totality of diagnostic performance studies was included. An example of search terms used was: (*Schistosoma haematobium*) AND (“LAMP”), depending on each different index test. Article mining in the references list of relevant reviews was also performed to ensure all studies were included in the search (Feleke et al., 2023) (Vaillant et al., 2024), (Zhou et al., 2025).

Following the removal of duplicates, two reviewers independently screened the remaining studies. Exclusions were done by title, then by abstract, and then by full-text screening, based on pre-determined inclusion/exclusion criteria. Any studies that had discrepancies between the reviewers were screened and resolved by consensus, or if necessary, independently resolved by a third reviewer. Suitable studies were uploaded into the Rayyan reference manager software (www.rayyan.ai) for the ease of independent review.

### Eligibility criteria of included studies

Studies were included if they analysed the diagnostic accuracy of POC or rapid field-applicable tests for the diagnosis of *S. haematobium* infections in individuals of all ages from endemic African countries. Non-species-specific indirect immunological tests were also included if the study used a reference test that diagnosed *S. haematobium* to confirm the diagnosis of UGS. Only studies in the English language were included in this systematic review and meta-analysis. Information about sensitivity and specificity or true-negatives (TNs), true-positives (TPs), false-positives (FPs), and false-negatives (FNs) was calculated from the data presented to produce contingency tables for diagnostic accuracy and receiver operating characteristic (ROC) analyses. Studies where this data could not be retrieved from the text were excluded. Trace positives were not considered in our review and were excluded from the accuracy calculations where possible. Primary observational studies were included if the results of an index test were compared to that of an appropriate laboratory-based reference standard. Case-control studies were included if cases and controls were sampled from the same patient population but were excluded if the control was from a healthy population from a non-endemic area. Proof-of-concept studies using artificially spiked urine were excluded, as well as commentaries, case studies, or editorials.

To provide an overall estimate of diagnostic accuracy, various laboratory-based reference tests were eligible, including standard microscopy, composite tests, PCR, or statistical models using Latent Class Analysis (LCA). Data about the reference test included sample preparation and the diagnostic threshold used to report a positive result. Bayesian modelling or any reference method where no test on biological samples was performed was excluded. In selecting eligible reference tests, parasitological methods were included, where urine sample concentration techniques (sedimentation, filtration, or centrifugation) and the use of single or double/triple microscopy were reported. Composite reference tests and LCA modelling using combined results from more than one test were eligible, provided that the type of tests used as comparators was reported. Studies that used the index test results as part of the composite reference test were excluded. For molecular-based reference tests, different types of PCR were accepted, including real-time PCR, the *Dra1-*based qPCR, and conventional PCR, provided that the DNA extraction and amplification techniques were reported. ELISA, Western Blot, or any laboratory test using biological samples from patients was included.

In selecting eligible index tests, POC tests were defined as those that can be conducted at or near the clinical site to facilitate timely and cost-effective decision-making (Drain et al., 2014). Rapid tests were defined as field versions of laboratory tests that use reagents that are not heat sensitive, utilising portable table-top equipment (such as mini centrifuging devices), and still ensure results are available within 2½ hours (World Health Organization, 2014). Relevant tests that meet the eligibility criteria of direct tests are antigen tests, molecular tests, and parasitological tests. The diagnostic methods include detecting *Schistosoma* antigens, schistosome DNA from the parasite itself, or viewing the eggs of *S. haematobium* in filtered urine. Rapid serological tests are also considered eligible for this review, as indirect tests, as their diagnostic value in new infections (Wami et al., 2014), and chronic infections is noted (González-Sanz et al., 2025). Urine reagent strips for haematuria and proteinuria have been excluded from this review as these tests serve as proxy tests and are unable to directly diagnose *S. haematobium*.

### Data extraction

Key characteristics of each study were extracted and summarised in a data extraction table in Microsoft Excel (Microsoft Corporation, 2022). The data extracted included study authors, publication year, journal name, country, prevalence of schistosomiasis in the population (low (<10%)/moderate (10%-50%)/high (>50%)), participant characteristics (including age, sex and method of participant selection), reference test standard used, index test (including name of manufacturer or equipment used, DNA extraction and amplification methods for molecular tests), index test sample, and threshold for positive results. The numbers of TPs, FNs, FPs, and FNs were entered into a 2 x 2 contingency table to calculate sensitivity and specificity measures manually, and these were compared to the published results for each study.

### Quality assessment

The methodological quality of the eligible studies was reviewed using a critical appraisal tool (Quality Assessment of Diagnostic Accuracy Studies 2, QUADAS-2). The tool was used to assess the risk of bias for diagnostic accuracy studies (Whiting et al., 2011). Four key domains of risk of bias and applicability to the research question assessed were patient selection, reference standard, index test, and flow and timing.

### Statistical analysis

From the data extraction table, studies were grouped according to index tests. Raw data from the extraction process were imported into the RevMan 5.3 software (training.cochrane.org) as recommended by the Cochrane Screening and Diagnostic Test Methods Group (SDTM) (Macaskill et al., 2010), to generate forest plots and ROC curves. TPs s, FNs, FPs, and FNs were entered into RevMan to calculate sensitivity and specificity measures with 95% confidence intervals for each study. A forest plot was generated for each index test group to visually report the sensitivity and specificity measures and confidence intervals. A ROC plot was generated from the data, producing a summary curve to visualise how the sensitivity and specificity measures of the tests performed against each other. The forest plots and ROC curves for each index test group were analysed graphically for heterogeneity, which was classified as low, medium, or high.

A meta-analysis was performed for all index tests appearing in three or more publications and which had no substantial heterogeneity. Tests with fewer than three studies were analysed narratively. Pooled estimates of sensitivity and specificity for each index test were calculated in STATA version 17 (STATA Corporation, 2015 statistical software: Release 14. College Station, Texas: StataCorp LP) or SAS version 9.3. (Copyright 2024 SAS Institute INC). The statistical model selected for the overall analysis for each test depended on how studies chose thresholds to determine positivity.

For tests that have a single common threshold, the bivariate random-effects model, as proposed by Reitsma was used for overall meta-analysis (Reitsma et al., 2005). The user-written command ‘metandi’ was utilized in STATA to fit the model and achieve pooled diagnostic accuracy results (Harbord and Whiting, 2009). A hierarchical summary (HSROC) model was utilised for index tests with variability among studies due to multiple positivity thresholds (Rutter and Gatsonis, 2001). HSROC provides an average diagnostic accuracy result to account for the within- and between-study variability among studies. The meta-analysis provided the final diagnostic accuracy result for each index test group.

### Investigations for heterogeneity

An exploratory analysis was performed for index test groups that visually displayed high heterogeneity in the forest plots and ROC analyses. An I2 statistic was not utilized due to the high variability in index and reference tests (Naaktgeboren et al., 2016). To determine if a factor/co-variate was associated with heterogeneity in test accuracy, forest plots and ROC curves were created in RevMan 5.3 for the following variables: prevalence setting, age-group, and reference test. These variables were chosen as possible reasons for heterogeneity according to the information provided in each study. ROC curves were generated for each of these three variables within each index test group and were examined visually to assess variations in accuracy for each variable. The heterogeneity was visually assessed as high, moderate, or low.

Index tests displaying high heterogeneity were further analysed using sub-group analysis techniques in STATA to determine actual sensitivity and specificity measures for each sub-group. For tests analysed with the bivariate random-effects model, we calculated sensitivity and specificity measures for each subgroup in STATA and then compared these results with the total pooled sensitivity and specificity measures. For tests analysed with the HSROC model, we investigated whether these covariates graphically affect the shape of the curve of the ROC plot generated separately for each covariate. Comparisons were made between the overall accuracy and subgroup accuracy for each index test. 

## Results

### Search results and eligible studies

**Figure 1:**
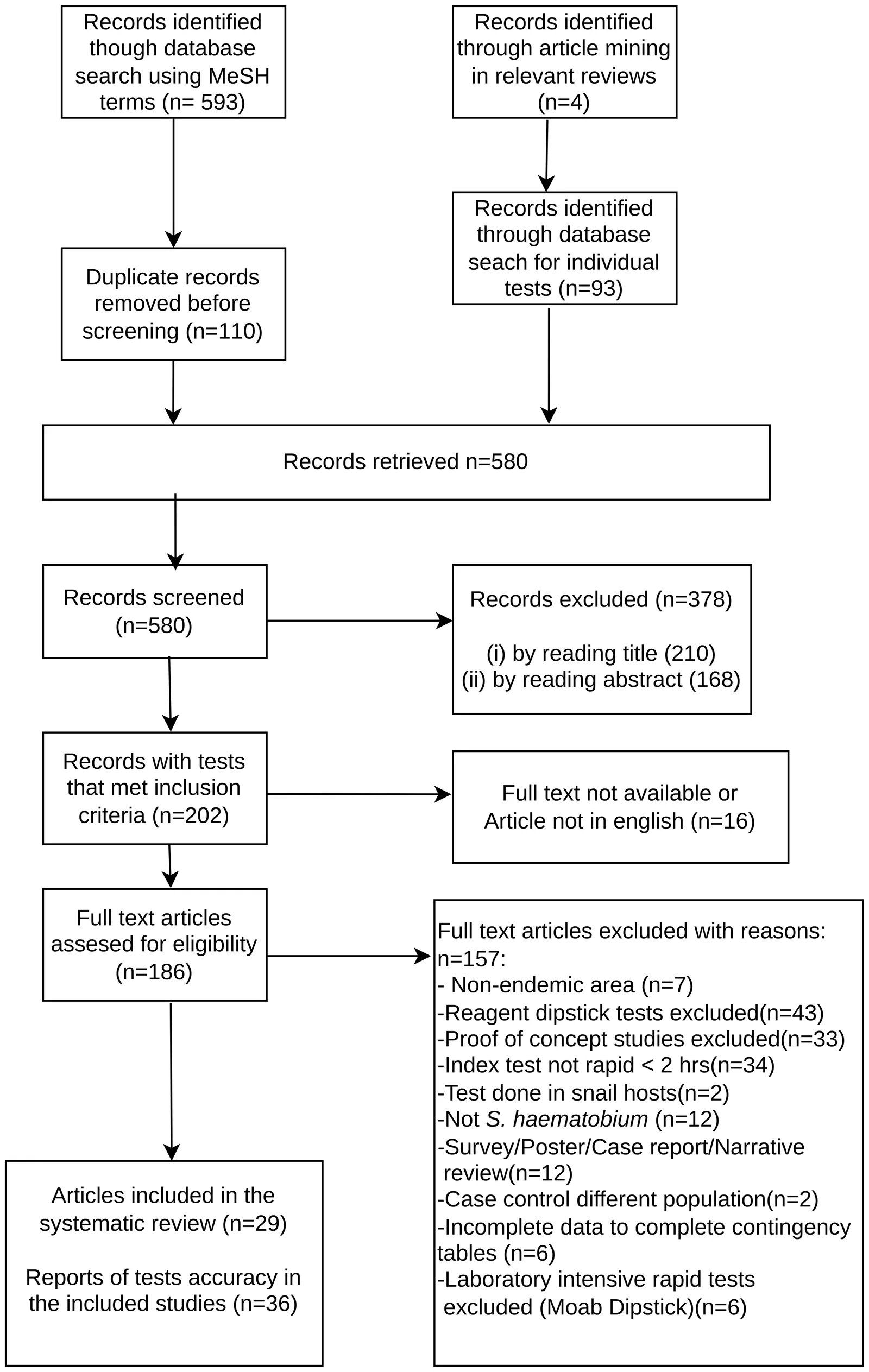
PRISMA flow diagram for the identification of studies included in this systematic review.

The search of databases identified 593 peer-reviewed studies on the diagnostic performance of POC/rapid tests for *S. haematobium.* A second index test-specific search produced 93 additional studies and 4 studies from citation screening in relevant reviews. After removing 110 duplicates, a total of 580 studies were screened independently by two reviewers who identified and excluded 378 non-eligible studies through title and abstract screening. A total of 202 studies were retained and assessed for eligibility. Twenty-nine studies were eligible according to our inclusion criteria, and data were extracted from these. Four studies analysed the diagnostic performance of more than one test; therefore, 36 evaluations from 29 studies were included. The 29 studies represent 9943 participants. The reasons for exclusion are reported in the PRISMA flowchart of the study selection process in **Figure 1**.

### Characteristics of included studies

The characteristics of the 29 included studies are presented in **Table 1**. All the studies included in the review were published from January 2000 to June 2026. Most of the studies, 26 (89.6%), were in field settings (village, school, or rural district) where study participants were from the general population in endemic areas. Two studies used banked samples from previous studies, and one study included patients in an outpatient clinic with suspected UGS.

**Table 1:** Characteristics of studies included in the systematic review and meta-analysis.

Author, year	Country	Participants	Study design	Setting	Prevalence of S. haematobium	Biological sample	Target	Index test (Thresholds)	Reference test
Serological tests									
(Yameny, 2019)	Egypt	Adults - 50 with UGS positive and 50 with UGS negative samples, age not stated	Case control	Endemic	Moderate (15%) Sh prevalent	Serum	Adult Worm Antigen	IHA (cutoff titre >1.160)	Single microscopy of centrifuged, filtered, or sedimented urine
(Zhang et al., 2020)	Zambia	148 school-aged children aged 7 to 15	Cross sectional	Endemic	High (61%) Co-endemic Sh/Sm	Serum	Adult Worm Antigen Soluble Egg Antigen	IHA (cutoff titre >1:1.20) DDIA (colour)	Double urine microscopy on filtered urine
(Houmsou et al., 2021)	Nigeria	374 participants from a rural area in Nigeria, all ages	Cross sectional	Endemic	Moderate 33% Sh prevalent area	Serum	Adult Worm Antigen	ICT-RDT (+test band)	Single microscopy of filtered urine
(Mudenda et al., 2022)	Zambia	430 adults residing in Siyavonga district, 18–50 years	Cross sectional	Endemic	Low 4% Co-endemic Sh/Sm	Serum	Adult Worm Antigen	ICT-RDT (+test band)	Single microscopy of filtered urine
(Nausch et al., 2014)	Zimbabwe	91 school children with egg counts for S. mansoni and/or S. haematobium, age not stated	Cross sectional	Endemic	High, 58% Sh prevalent area	Serum	Cercarial transformation fluid (SmCTF)	SmCFT-RDT (+test band)	Single microscopy of filtered urine
(Coulibaly et al., 2013)	Cote Ivoire	118 preschool children (3–6 years)	Cross sectional	Endemic	Low, 5.1% Co-endemic Sh/Sm	Finger-prick blood	Cercarial transformation fluid (SmCTF)	SmCFT-RDT (+test band)	Double microscopy of filtered urine
Antigen Tests									
(Stothard et al., 2006)	Zanzibar Tanzania	50 school children - 25 positive, 25 negative for S. haematobium, age not stated	Case control	Endemic	High, 73% Sh prevalent area	Urine	Cathodic antigen	POC-CCA (+test band)	Single microscopy of filtered urine
(Obeng et al., 2008)	Ghana	152 school children, 5–16 years	Case control	Endemic	Low, 0-20% Co-endemic Sh/Sm	Urine	Cathodic antigen	POC-CCA (+test band)	Single microscopy of filtered urine
(Ayele et al., 2008)	Ethiopia	206 school children, 4–21 years	Cross sectional	Endemic	High 47% Sh prevalent area	Urine	Cathodic antigen	POC-CCA (+test band)	Single microscopy of filtered urine
(Ashton et al., 2011)	Sudan	373 children, 5–16 years	Cross sectional	Endemic	Moderate, 26% Co-endemic Sh/Sm	Urine	Cathodic antigen	POC-CCA (+test band)	Single microscopy of filtered urine
(El-Ghareeb et al., 2016)	Egypt	96 school children, 5–12 years	Cross sectional	Endemic Post MDA	Low, 5.3% Sh prevalent area	Urine	Cathodic antigen	POC-CCA (+test band)	Single microscopy of filtered urine
(Sanneh et al., 2017)	The Gambia	1954 school children, 7–14 years	Cross sectional	Endemic	Low, 10% Sh prevalent area	Urine	Cathodic antigen	POC-CCA (+test band)	Single microscopy of filtered urine
(Rubaba et al., 2018)	South Africa	420 school children, 10–15 years	Cross sectional	Endemic	Moderate, 40% Sh prevalent area	Urine	Cathodic antigen	POC-CCA (+test band)	Single microscopy of filtered urine
(Robinson et al., 2021)	Madagascar	500 adults and children, age not stated	Cross sectional	Endemic	High, 47% Co-endemic Sh/Sm	Urine	Cathodic antigen	POC-CCA (+test band)	Single microscopy of filtered urine
(Sheele et al., 2013)	Kenya	160 school children with recent blood in urine, 8–17 years	Cross sectional	Endemic	High 49% Sh prevalent area	Urine	Eggs	RDT-Sh (colour change)	Single microscopy of filtered urine
Nucleic acid amplification tests (NAAT’s)									
(Gandasegui et al., 2018) (b)	Angola	172 school children, aged 8–15 years	Cross sectional	Endemic	High, 50.6% Sh prevalent area	Urine	DNA (Ribosomal intergenic spacer)	Sh-LAMP Conventional method (fluorescence)	Single microscopy of filtered urine
(Gandasegui et al., 2018) (a)	Angola	172 school children, aged 8–15 years	Cross sectional	Endemic	High, 50.6% Sh prevalent area	Urine	DNA (Ribosomal intergenic spacer)	Sh-LAMP Rapid-Heat LAMPellet method (fluorescence)	Single microscopy of filtered urine
(Bayoumi et al., 2016)	Egypt	69 adults with suspected schistosomiasis at an outpatient clinic	Cross sectional	Endemic	Moderate, 44.9% Co-endemic Sh/Sm	Urine	DNA (Ribosomal intergenic spacer)	Sh-LAMP Conventional method (fluorescence)	Single microscopy of filtered and centrifuged urine
(Lodh et al., 2017)	Ghana	86 participants, aged 5–26 years	Cross sectional	Endemic	High, 81% Co-endemic Sh/Sm	Urine	DNA (Ribosomal intergenic spacer)	Sh-LAMP Conventional method (fluorescence)	PCR Urine
(van Bergen et al., 2024)	Madagascar	Archived DNA samples extracted from 125 vaginal mucosal swabs from women aged 15–35 years.	Cross Sectional	Endemic	High, 67% Sh prevalent area	Cervico-vaginal fluid	DNA (Ribosomal intergenic spacer)	Sh-LAMP Conventional method (fluorescence)	qPCR of cervical lavage samples
(Archer et al., 2022)	Zambia	527 non-pregnant, sexually active women, 18–35 years.	Cross sectional	Endemic	Low, 2.5% Sh prevalent area	Cervico-vaginal fluid	DNA (Sh-Dra1 repeat sequence)	RPA-Sh (200mV)	PCR of cervical lavage samples
(Frimpong et al., 2021)	Ghana	1290 participants resident in Kumasi, from whom 69 positive and 66 negative urine samples were chosen, all ages	Case Control	Endemic	Low, 5.3% Co-endemic Sh/Sm	Urine	DNA (Sh-Dra1 repeat sequence)	RPA-Sh (900mV)	Composite reference (RT-PCR of urine and single microscopy of centrifuged urine)
(Archer et al., 2020)	Zanzibar Tanzania	168 school children - 100 UGS positive and 68 random controls, 9–12 years	Case control	Endemic	Low, <5% Sh prevalent elimination setting	Urine	DNA (Sh-Dra1 repeat sequence)	RPA-Sh (200mV)	Single microscopy of filtered urine
Parasitological tests									
(Armstrong et al., 2022)	Ghana and Côte d’Ivoire	205 School-aged children, of which 63 were from Ghana and 142 from Côte d’Ivoire	Cross sectional	Endemic	High 55% Sh prevalent area	Urine	Eggs	Portable microscope: Schistoscope AI	Conventional single microscopy of filtered urine
(Meulah et al., 2024) (a)	Gabon	339 school-aged children (aged 7-13) and adults	Cross sectional	Endemic	Moderate 30% Sh prevalent area	Urine	Eggs	Portable microscope: Schistoscope AI	Conventional single microscopy of filtered urine
(Meulah et al., 2024) (b)	Gabon	339 school-aged children (aged 7-13) and adults	Cross Sectional	Endemic	Moderate 30% Sh prevalent area	Urine	Eggs	Portable microscope: Schistoscope AI	Composite reference (Single microscopy, rt-PCR urine, UCP-LFCAA urine)
(Meulah et al., 2024) (c)	Gabon	Biobanked urine samples from 798 school-aged children, adults and pregnant women	Cross Sectional	Endemic	Moderate 30% Sh prevalent area	Urine	Eggs	Portable microscope: Schistoscope AI	Conventional single microscopy of filtered urine
(Meulah et al., 2024) (d)	Gabon	Biobanked urine samples from 349 school-aged children, adults and pregnant women	Cross sectional	Endemic	Moderate 30% Sh prevalent area	Urine	Eggs	Portable microscope: Schistoscope AI	Conventional single microscopy of filtered urine
(Meulah et al., 2024) (e)	Gabon	Biobanked urine samples from 349 school-aged children, adults and pregnant women	Cross Sectional	Endemic	Moderate 30% Sh prevalent area	Urine	Eggs	Portable microscope: Schistoscope AI	Composite reference (Single microscopy, rt-PCR urine, UCP-LFCAA urine)
(Meulah et al., 2022)	Nigeria	487 pre-school, school-aged children, and adults	Cross sectional	Endemic	High 49% Sh prevalent area	Urine	Eggs	Portable microscope: Schistoscope automated 5.0	Conventional single microscopy of homogenised filtered urine
(Bogoch et al., 2017)	Ghana	223 school children who provided 60 urine samples, 7–16 years	Cross sectional	Endemic	High, 73% Sh prevalent area	Urine	Eggs	Mobile-phone handheld microscope	Conventional single microscopy of centrifuged urine
(Ephraim et al., 2015)	Ghana	School children who provided 49 urine samples, 7–13 years	Cross sectional	Endemic	High, 69% Sh prevalent area	Urine	Eggs	Mobile-phone handheld microscope (Cellscope)	Conventional single microscopy of centrifuged urine
(Coulibaly et al., 2016) (a)	Cote d’Ivoire	226 children and young adults aged 6–19 years	Cross sectional	Endemic	Moderate, 40% Co-endemic Sh/Sm	Urine	Eggs	Mobile-phone handheld microscope (Newton NM1)	Conventional single microscopy of filtered urine
(Coulibaly et al., 2016) (b)	Cote d’Ivoire	226 children and young adults aged 6–19 years.	Cross sectional	Endemic	Moderate, 40% Co-endemic Sh/Sm	Urine	Eggs	Mobile-phone handheld microscope (Cellscope)	Conventional single microscopy of filtered urine
(Coulibaly et al., 2023)	Cote d’Ivoire	170 children aged 5–14 years	Cross sectional	Endemic	Moderate 20% Co-endemic Sh/Sm	Urine	Eggs	Portable microscope: Schistoscope AI	Conventional single microscopy of filtered urine

Sh: S.haematobium, Sm: S.mansoni.

The median prevalence of *S. haematobium* infection was 30.7% (range 2.5% - 73.0%). In **Table 1**, prevalence is defined as high (infection diagnosed in >51% of school-aged children (SAC)), moderate (>10% and <50% in SAC), and low (<10% in SAC). In 6 studies (20.6%), participants in co-endemic populations were tested for both *S. haematobium* and *S. mansoni* using non–species-specific antigen or antibody assays, although the results have been presented for *S. haematobium* only. Fourteen studies focused on diagnostic tests that specifically identify evidence of *S. haematobium* for the diagnosis of UGS only. Most of the studies were cross-sectional or cohort studies (86.2%), either using urine/serum samples from previous studies or collecting urine samples from participants in field studies. Four were case-control studies comparing serum samples from individuals with UGS and without UGS from the same population.

Nine rapid diagnostic testing techniques were identified as eligible index tests through the systematic search for diagnostic accuracy studies, as presented in **Table 2**. Four of these index tests were entered into the meta-analysis, as five index tests had two or less diagnostic accuracy studies published and therefore were analysed narratively. Only the circulating cathodic antigen test (POC-CCA) is considered a POC test because it can be conducted at or near the clinical site (Drain et al., 2014). The other eight tests are considered rapid as they use field-based kits and minimal portable laboratory equipment, with results available within 2½ hours.

**Table 2:** Diagnostic tests identified as eligible rapid and POC index tests through a systematic search of diagnostic accuracy studies and included in this review.

Test	Test name and description	Target	Rapid /POC	No. of reports	Reason for consideration
LAMP	Loop Mediated Isothermal Amplification LAMP is a nucleic acid amplification test (NAAT) that amplifies schistosome DNA under isothermal conditions (60–65 °C) Sample required: Urine Species specific for S. haematobium.	DNA (Ribosomal intergenic spacer)	Rapid	5	Time to result: 10 mins for DNA extraction, 1–2 hours for DNA amplification (Gandasegui et al., 2018). Field applicability: Minimal equipment needed, centrifuge, heat block, commercial LAMP reagents
Sh-RPA	Recombinase polymerase amplification The Sh-RPA assay is a NAAT that amplifies schistosome DNA at constant isothermal temperatures (37-42 °C). Sample required: Urine Species specific for S. haematobium	DNA (Sh-Dra1 repeat sequence)	Rapid	3	Time to result: Approximately 1 hour: 10 mins for DNA extraction and amplification, 15 mins for RPA reaction at 42°C, 10 mins for Fluorometer reading (Archer et al., 2022) Field applicability: Minimal equipment needed; the RPA reaction is initiated in test tubes with hand shaking or centrifuging. A portable testing device measures fluorescence.
IHA	Indirect haemagglutination assay Serological test detecting antibodies in serum to worm antigen using haemagglutination techniques. Sample required: Serum Species specific: No. Cannot distinguish between S. haematobium and S. mansoni.	Adult Worm Antigen	Rapid	2	Time to result: Approx 2 ½ hours: 10 minutes for obtaining serum, 10 minutes for the agglutination step, 2 hours for incubation and result (Yameny, 2019). Field applicability: Simple test requiring only basic laboratory equipment: table-top centrifuge, reagents from the IHAT Fumouze kit.
ICT-RDT	Immunochromatographic- rapid diagnostic test Serological test detecting antibodies to Schistosoma spp antigen in serum, using a lateral flow cassette. Sample required: Serum Species specific: No. Cannot distinguish between S. haematobium and S. mansoni.	Adult Worm Antigen	Rapid	2	Time to result: 20–30 minutes. 10 minutes for obtaining serum, 10 minutes for the test (Mudenda et al., 2022). Field applicability: simple to use, only a tabletop centrifuge is required to attain serum
SmCTF-RDT	S.mansoni-cercarial transformation fluid- RDT Serological test detecting antibodies to S.mansoni cercarial transformation fluid (SmCTF) in serum. Sample required: Serum or finger-prick blood Species specific: No. Cannot distinguish between S. haematobium and S. mansoni.	Cercarial transformation fluid	Rapid	2	Time to result: 15 to 20 minutes. 10 minutes to obtain serum, 10 minutes for the test (Nausch et al., 2014). Field applicability: simple to use, only a tabletop centrifuge is required to attain serum
DDIA	Dipstick Dye Immuno Assay Serological test detecting antibodies to soluble egg antigen (SEA) in serum. Sample required: Serum Species specific: No. Cannot distinguish between S. haematobium and S. mansoni.	Soluble Egg Antigen	Rapid	1	Time to result: 20 minutes. Field applicability: A table-top centrifuge is required to attain serum, PVC well, micropipette, dipstick (Zhang et al., 2020).
RDT-Sh	Rapid Diagnostic Test- S. haematobium An antigen test designed to detect eggs in urine by antibody-antigen reaction on the egg surface. Sample required: Urine Species specific for S. haematobium	Eggs	Rapid	1	Time to result: 20 minutes Field applicability: Yes, simple and cheap, no laboratory needed, only a syringe and filter coupling device, reagent, and cup (Sheele et al., 2013)
POC-CCA	Point of Care- Circulating Cathodic antigen Urine- based lateral flow antigen test cassette that detects the circulating cathodic antigen (CCA) from the adult Schistosoma worm in urine, Sample required: Urine Species specific: No. Cannot distinguish between S. haematobium and S. mansoni.	Cathodic Antigen	POC	8	Time to result: 20 minutes. Field applicability: Yes, simple to use; 1 drop of urine and a subsequent drop of buffer (old test version only). No laboratory equipment needed
Portable/mobile phone microscopy	Three portable microscopes using a mobile phone or digital/AI technology are included: -The Cellscope: attached to a mobile phone - The Newton 1: attached to a mobile phone -The Schistoscope, which is a digital automated microscope, is attached to a laptop. Sample required: Urine Species specific for S. haematobium	Eggs	Rapid	12	Time to result: 45 mins to 1 hour Field applicability: The devices can be used without laboratory equipment as stand-alone devices
TOTAL				36	

The indirect haemagglutination assay (IHA) and the immuno-chromatographic rapid diagnostic tests (ICT-RDT), the *S. mansoni*-cercarial transformation fluid-RDT (SmCTF-RDT), and the dipstick dye immunoassay (DDIA) are non-species-specific serological screening tests that detect antibodies in serum (Yameny, 2019) (Houmsou et al., 2021) (Nausch et al., 2014). These assays require blood specimens and the availability of portable centrifuges to obtain serum in the field. The POC-CCA detects circulating cathodic antigen (CCA) in urine but is also not species-specific, with rapid results in under 30 minutes using a simple urine-based lateral flow cassette. Non-species-specific tests are excellent screening tests for both urinary and intestinal schistosomiasis. However, in areas where both species exist, these tests need to be used with caution if the sensitivity of the test for either species differs, to prevent false negatives. The POC-CCA is known to diagnose *S. mansoni*, but has poor accuracy for *S. haematobium* (Ochodo et al., 2015; Rubaba et al., 2018), resulting in underdiagnosis in co-endemic areas. Eleven included studies (37.9%) were performed in areas where both *S. haematobium* and *S. mansoni* were prevalent, or in populations from co-endemic areas, further highlighting the need for species-specific tests, or tests with high accuracy for both species of *Schistosoma*.

One study, the RDT- *S. haematobium* (RDT-Sh), describes a novel, cheap, and simple method to detect antibodies in urine samples using a syringe, filter, and anti-IgG for detecting schistosome eggs specific for *S. haematobium* (Sheele et al., 2013).

With recent advances in molecular DNA-based field-applicable testing, the loop-mediated isothermal amplification (LAMP) and the recombinase polymerase amplification (*Sh*-RPA) tests are both being developed to be rapid diagnostic assays to detect schistosome DNA fragments in urine samples. The LAMP and RPA tests are *Schistosoma* species-specific tests that can distinguish between *S. haematobium* and *S. mansoni*. In these tests, the DNA extraction and amplification reagents used do not require cold chain logistics and minimal equipment is required (Gandasegui et al., 2015) (Archer et al., 2020). DNA extraction requires centrifuging of urine to obtain a pellet of urinary sediment, and the amplification reaction requires incubation at 65°C using a heating block. Both tests are rapid tests using minimal laboratory equipment, with results obtainable within 2½ hours in field settings.

Portable/mobile-phone microscopy using portable devices connected to a mobile phone are also species-specific tests that can identify *S. haematobium* eggs in filtered or centrifuged urine. The Schistoscope was categorized with the portable/mobile-phone microscopes, as this is an automated microscope developed specifically for low-resource settings (Meulah et al., 2022).

In **Table 1**, test thresholds to determine positivity are provided in brackets next to each index test so that variable test thresholds can be identified for each index test group. Lateral flow cassette tests which have a colour change for positive results are indicated as + test band. Tests are categorized as serological, antigen, NAAT (molecular), and parasitological.

It can be noted that studies conducted before 2020 used single microscopy as a reference test, and those after 2020 used improved reference tests such as PCR, latent class analysis, or double microscopy. In high-prevalence endemic areas, microscopy can be considered an appropriate reference test (World Health Organization, 2022). However, 6 studies (20.6%) used microscopy of urine as a reference test in low/moderate prevalence settings, which could cause bias in the overall results.

### Appraisal of risk of bias and applicability

**Figure 2:**
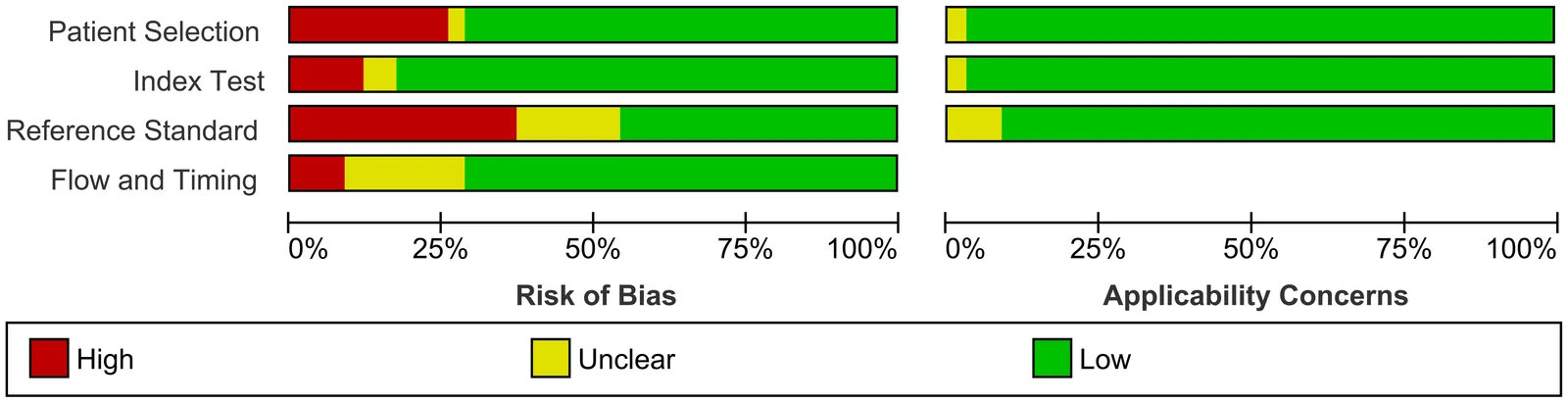
Risk of bias and applicability concerns for the studies reviewed (Note: the authors’ judgements about each domain are presented as percentages across included studies.).

The QUADAS-2 risk assessment results are presented in **Figure 2** and further elaborated in **Supplementary Additional File 2**. The overall methodological quality of the studies was rated as moderate. Methods of participant selection were adequately described; however, eight studies (25%) chose known positive and negative samples instead of randomly selected samples and were therefore classed as high risk of bias for patient selection. Selection bias can be described in one of the studies, where participants were chosen from patients who attended health clinics and were not representative of the general population. In over 75% of the studies, random selection of participants was conducted.

Over 85% of the studies appropriately reported the index test, described how the tests were conducted, ensured that results from each test were blinded to prevent bias, and explained how the results were interpreted. In the index test domain, risk of bias relates to tests that are not blinded. The accuracy of the reference tests differed by reference test type and prevalence of infection. In the reference test domain, more than 40% of studies in low and moderate prevalence settings used the inaccurate single microscopy test as the reference, resulting in a high risk of bias. In the flow and timing domain, approximately 10% of the studies stated that the interval between the index and reference tests may have caused deterioration of sample quality, but most ensured that samples were frozen if the reference test was not performed on-site.

In terms of applicability concerns, most (90%) of the studies were aligned with the current study’s research question.

### Diagnostic performance

Forest plots for the included index tests with two or more studies are presented in **Figure 4**, allowing a visual comparison of each index test group. The sensitivity and specificity measures for each study in an index test group are plotted with 95% confidence intervals. The results of the final meta-analysis are presented in **Table 3** as an overall summary point for the sensitivity and specificity of each included index test. Bivariate modelling was performed for POC-CCA, portable/mobile-phone microscopy, and LAMP, as these tests use the same threshold for determining positivity. HSROC modelling was used for the RPA test, as multiple thresholds were used for determining positivity in the included studies.

**Table 3:** Pooled sensitivity and specificity results for rapid and POC index tests that diagnose urogenital schistosomiasis for individuals infected in African settings.

Index test type	No. of accuracy records	Pooled number of participants	No. with schistosomiasis (%)	Sensitivity % (95% CI)	Specificity % (95% CI)
Serological tests					
IHA	2	198	139 (70.2)	59.5% (35.8-79.5)	76.1% (65.1-84.5)
ICT-RDT	2	804	219 (27.2)	98.6% (77.0-99.9)	47.7% (27.7-69.5)
RDT-Sh	1	150	78 (52.0)	90.0% (81.0-95.0)	54.0% (42.0-65.0)
SmCTF-RDT	2	209	77 (36.8)	98.4% (7.70-99.9)	36.7% (27.4-47.6)
DDIA	1	146	89 (60.9)	60.0% (49.0-70.0)	61.0% (48.0-74.0)
Antigen Tests					
POC-CCA	8	3751	887 (23.6)	49.8% (25.4-73.4)	77.6% (65.1-86.5)
NAAT tests					
LAMP	5	542	230 (42.4)	94.7% (67.2-99.3)	66.4% (46.99-81.6)
RPA	3	830	235 (28.3)	94.4% (90.0-96.0)	96.6% (96.0-99.0)
Parasitological tests					
Portable or mobile microscopy	12	2497	805 (32.2)	81.5% (72.6-88.0)	91.4% (86.5-94.6)

IHA: Indirect haemagglutination assay, ICT-RDT: Immunochromatographic rapid diagnostic test, SmCTF-RDT: *S. mansoni*-cercarial transformation fluid- RDT, DDIA: Dipstick dye immunoassay, RDT-Sh: Rapid Diagnostic Test- *S. haematobium*, POC-CCA: Point of Care- Circulating Cathodic antigen, LAMP: Loop Mediated Isothermal Amplification, RPA: Recombinase Polymerase Amplification.

In the final meta-analysis, the molecular test RPA had the highest overall accuracy (sensitivity, 94.4%; specificity, 96.6%), displaying the best trade-off between sensitivity and specificity. Based on this meta-analysis, portable/mobile-phone microscopy (sensitivity, 81.5%; specificity, 91.4%) had the second-highest diagnostic accuracy. LAMP (sensitivity, 94.7%; specificity, 66.4%) and the serological screening test ICT-RDT (sensitivity, 98.6%; specificity, 47.7%) come close in accuracy for *S. haematobium* diagnosis, where both are sensitive tests but lack specificity. Tests with moderate accuracy are the SmCTF-RDT and RDT-Sh serological tests, where further research with improved reference tests could provide answers for their use as rapid tests. Our overall results indicate that the IHA (sensitivity, 59.5%; specificity, 76.1%), DDIA (sensitivity, 60.0%; specificity, 61.0%), and POC-CCA (sensitivity, 48.9%; specificity, 77.3%) index tests have low diagnostic accuracy for *S. haematobium* and may not be suitable for prevalence mapping or estimation of infection. These tests miss infections that are identified by laboratory-based tests and are therefore not useful in practice.

### Summary ROC curve for all included index tests

**Figure 3:**
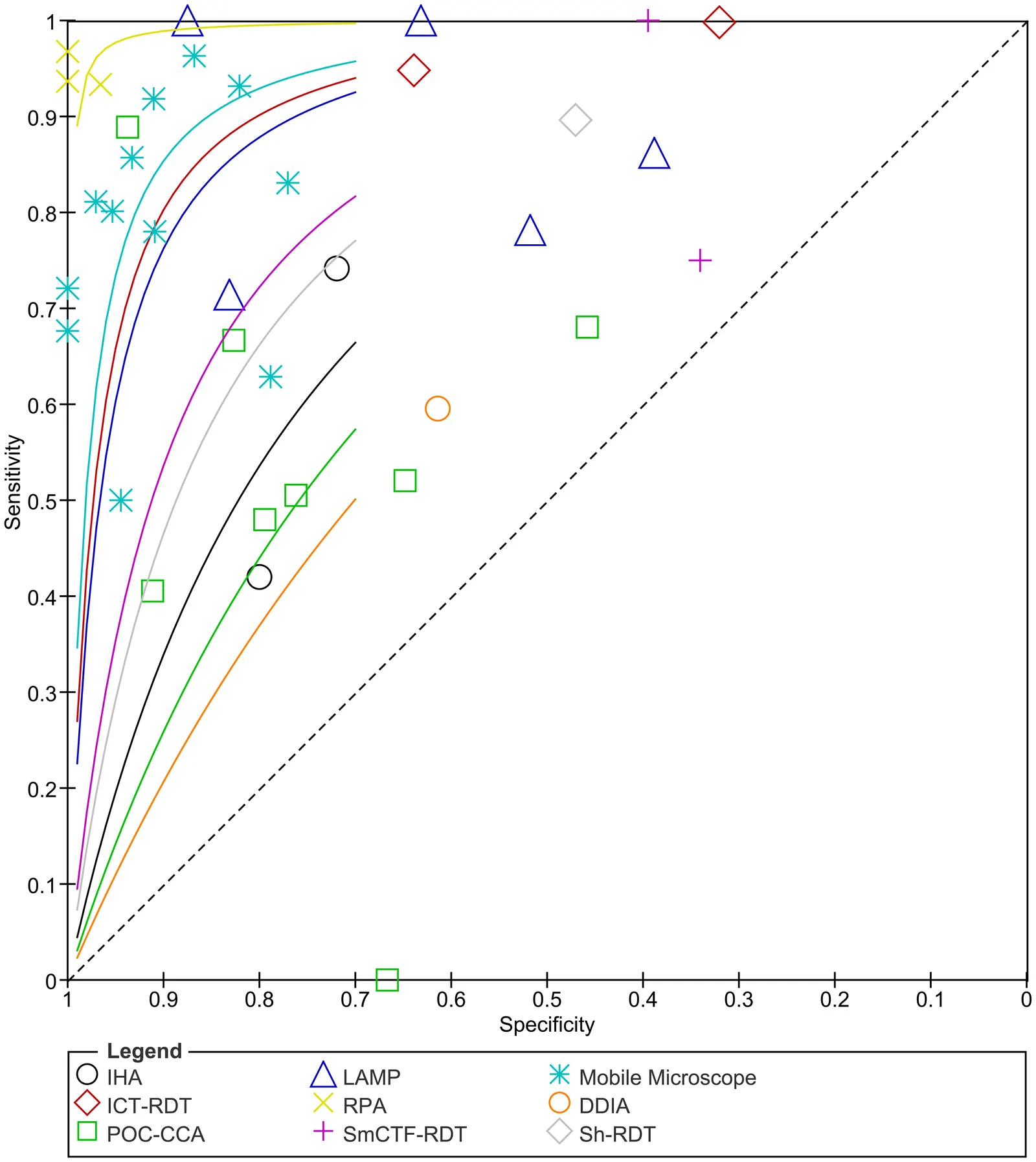
Summary ROC curves for all included index tests.

To enable comparisons, the summary ROC curves for all index tests are presented in a single graph in **Figure 3**. Tests with higher accuracy are in the top left corner. This shows that the molecular test RPA has the highest overall accuracy. Portable/mobile-phone microscopy, ICT-RDT, and LAMP are grouped as the second-highest diagnostic accuracy, while POC-CCA and DDIA have the poorest diagnostic accuracy. 

**Figure 4:**
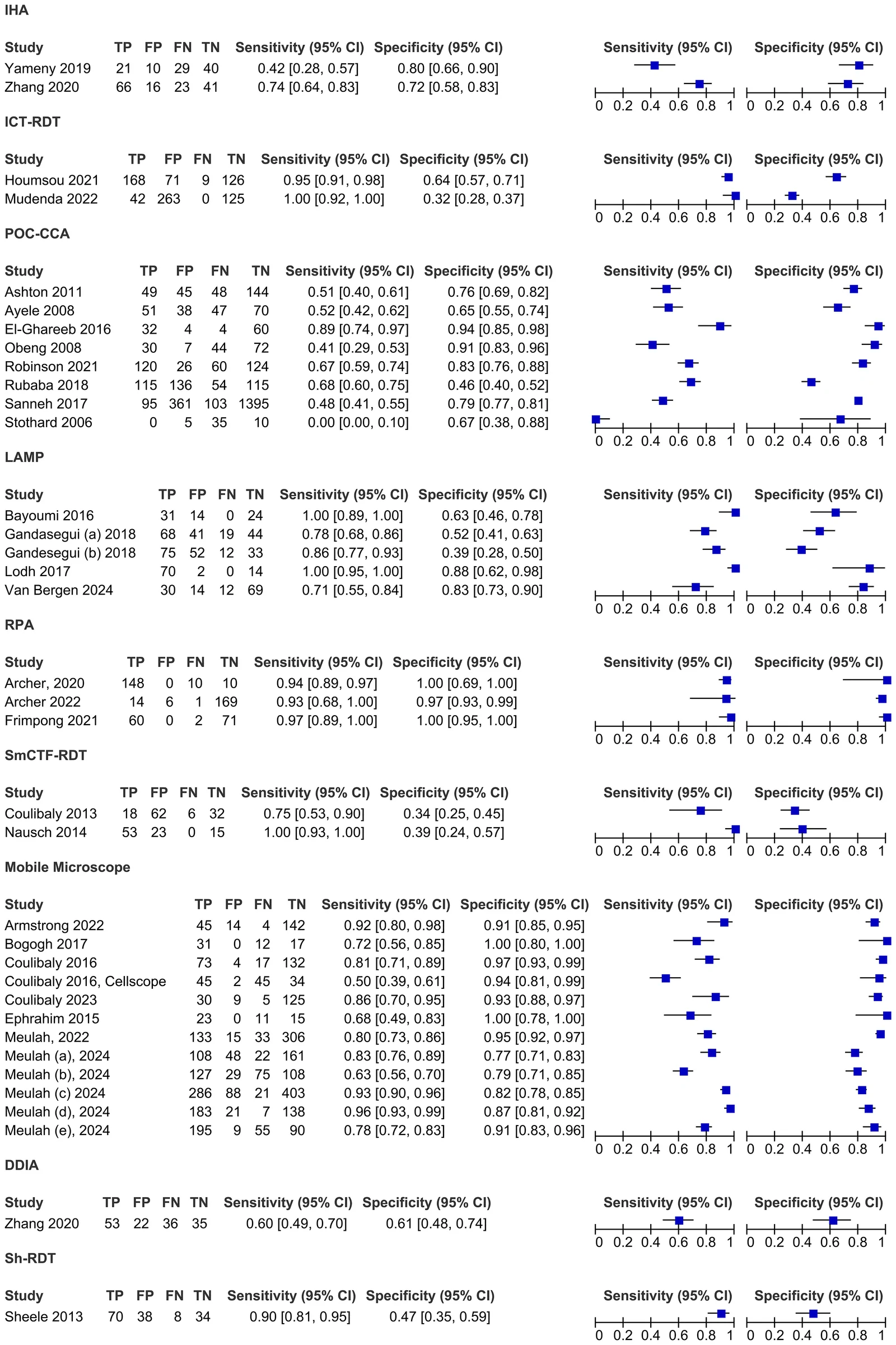
Forest Plot of included studies grouped according to the index test.

### Exploration of heterogeneity

The forest plots (**Figure 4**) and the individual SROC curves for each index test group (**Supplementary Additional File 3**) show visually that there is high heterogeneity among the studies for POC-CCA and LAMP, and moderate heterogeneity for RPA and portable/mobile-phone microscopes. To further determine possible reasons for heterogeneity, an exploratory analysis using forest plots and SROC plots (**Supplementary Additional File 4**) and a subgroup meta-analysis (**Table 4**) were performed for the covariates: prevalence setting, reference standard, and age group.

**Table 4:** Subgroup analysis for individual index tests comparing overall accuracy results with subgroup accuracy results.

Co-variate	Sub-group (records)	Participants(n)	Sensitivity (%) (95% CI)	Specificity (%) (95% CI)
Antigen tests				
POC-CCA: Overall accuracy (8 records)		N=3751	49.8 (25.4-73.4)	77.6 (65.1-86.5)
Prevalence setting high	Endemic high Sh prevalent (3)	676	18.8 (0.0- 91.7)	59.9 (43.4-74.4)
	Endemic high co-endemic Sh/Sm (1)	500	67.0 (59.0-74.0)	83.0 (76.0-88.0)
Prevalence setting low	Endemic low Sh prevalent (2)	2050	72.6 (34.7-92.9)	87.6 (72.3-95.0)
	Endemic low co-endemic Sh/Sm (2)	525	45.6 (36.0-55.5)	84.3 (70.0-92.5)
Age group	Adults>18 years (1)	500	67.0 (59.0-74.0)	83.0 (76.0-88.0)
	Children (7)	3251	45.9 (19.8-7.5)	76.8 (62.-86.9)
NAAT tests				
LAMP: Overall accuracy (5 records)		(N = 542)	94.7 (67.2-99.3)	66.4 (46.99-81.6)
Reference standard	PCR (2)	211	94.3 (50.0-99.6)	73.7 (41.5-91.7)
	Single microscopy (3)	331	91.4 (64.5-98.4)	50.7 (39.0-62.4)
Age group	Adults& children (3)	280	98.0 (47.6-99.9)	70.5 (46.7-86.7)
	School Children only (2)	262	82.4 (74.1-88.4)	45.2 (35.3-55.5)
Prevalence setting	Endemic high Sh prevalent (3)	469	79.0 (69.3-86.3)	59.6 (35.6-79.8)
	Endemic high co-endemic Sh/Sm (2)	155	99.4 (46.6-99.9)	63.8 (36.9-84.2)
Parasitological tests				
Portable microscopy: Overall accuracy (12 records)		(N = 2497)	81.5 (72.6-88.0)	91.4 (86.5-94.6)
Device type	Portable microscope (2) Cellscope (2) Schistoscope (8)	449 275 1773	76.5 (68.2-82.3) 59.0 (47.3-73.5) 86.3 (77.7-91.9)	98.5 (97.5-99.2) 99.5 (97.5-99.2) 88.1 (82.8-92.0)

Sh, S. haematobium; Sm, S. mansoni.

For POC-CCA, the low-prevalence setting in an *S. haematobium-*prevalent area had improved sensitivity (72.6% vs 49.8%), and poor sensitivity results were found for high *Sh*-prevalent areas (18.8%). A high *S. haematobium* prevalence is therefore not suitable for diagnosis using the POC-CAA test due to many false positives, and possibly due to cross reactions with *S. mansoni*. Single microscopy was used as a reference for all the POC-CCA studies, which may have caused bias in the specificity results of 3 of the POC-CCA studies that demonstrate low *Sh* prevalence.

For LAMP, the quality of the reference test affects the quality of the results: specificity improves (73.7% vs 66.4%) when PCR is used as the reference test, although sensitivity does not change much. The PCR reference standards show superior diagnostic performance compared to single microscopy, implying that accuracy is inaccurately portrayed by the usual gold standard using single microscopy of filtered urine.

Age group also caused heterogeneity in the results for LAMP and POC-CCA where a combined population of all ages had a better sensitivity than school-aged children (98.0% vs 82.4%) and (67.0% vs 45.9%), respectively.

The Schistoscope revealed the highest sensitivity (86.3%) of the three portable microscopes, where the Cellscope and mobile-phone attached microscope had lower sensitivities (59.0% and 76.5%, respectively), but improved specificities. The studies were all performed in high-moderate endemic settings, and further validation could determine the accuracy of portable microscopes in low-prevalence settings.

## Discussion

This systematic review sought to identify a rapid test with high sensitivity and specificity to improve prevalence mapping and elimination-of-transmission testing, replacing microscopy in integrated control strategies in low-resource and rural areas. The results of this review can support the choice of diagnostic test in endemic areas with poor laboratory infrastructure and collate the available evidence on the accuracy measures of rapid field-based diagnostic tests for the detection of *S. haematobium*. Previous systematic and narrative reviews have focused on testing and screening both *S. mansoni* and *S. haematobium* (Vaillant et al., 2024) (Rivera et al., 2024) and not specifically for field-based testing in low-resource areas. Recent reviews have been published focusing on individual tests, for example, the LAMP assay in low-endemic areas (Zhou et al., 2025), and AI-assisted diagnostic tools (Desale et al., 2026). This review provides a broader synthesis of multiple rapid and field-deployable diagnostic tools in low-resource areas and thereby offers a comparison between the performance of various rapid field-based tests.

A key finding was that the field-based molecular tests RPA and LAMP are the most suitable tests for prevalence estimation of active infections in both high and low prevalence settings and for various stages of chronicity. RPA has the highest accuracy in low-intensity and chronic infections and is also most useful for the estimation of the elimination of transmission of *S. haematobium*. These tests are still in development and require portable testing devices to obtain results from urine samples but show great promise for use as test-and-treat tools in low-resource areas with field laboratories. Considering the REASSURED criteria (Land et al., 2019) as the benchmark to assess POC and rapid tests, the RPA and LAMP offer an affordable, sensitive, and specific solution.

The portable/mobile-phone microscopy tests using handheld cell phone devices and AI-assisted microscopy, such as the Schistoscope (sensitivity, 81.4%, specificity, 91.4%) have great value in areas where skilled microscopists are unavailable and show promise in the automation of egg detection using microscopy of urine.

Another finding was that the ICT-RDT and SmCTF-RDT serological tests have high sensitivity (98.6% and 98.4%, respectively) in all prevalence settings. However, these tests require trained phlebotomists to draw blood and portable centrifuges to separate serum from whole blood. The SmCTF-RDT can also be performed using finger-prick blood, which is far easier to obtain. The low specificity of the ICT-RDT and SmCTF-RDT tests (47.7% and 36.7%, respectively) was due to reference test bias, as the comparator tests were microscopy in a low-prevalence area. These serological tests do not differentiate between active or past infection, and between *S. haematobium* and *S mansoni.* They are therefore excellent screening tests for travellers and migrants and in areas of co-endemicity, detecting infections with either species of schistosomiasis. Both species are treated with Praziquantel, which can be administered in the early stage of infection (Zwang and Olliaro, 2014).

Our review also determined that two tests have low sensitivity and, therefore lower diagnostic accuracy than conventional microscopy. These are IHA (sensitivity, 59.5%, specificity, 76.1%), and DDIA serological test (sensitivity, 60.0%, specificity, 61.0%). These tests are moderately sensitive and therefore not suitable for prevalence mapping or estimation of infection.

The findings of this study agree with those of previous reviews (Ochodo et al., 2015) (Stothard et al., 2006) that the POC-CCA lateral flow test, used extensively for *S. mansoni* diagnosis in Africa, has low diagnostic accuracy for *S. haematobium* (sensitivity, 49.8%, specificity, 77.6%). The test results in false negatives and misses many *S. haematobium* infections detected using laboratory-based tests. There was a high heterogeneity for POC-CCA results, where the *S. haematobium-*prevalent areas had low sensitivity, even if infection prevalence is high. As the POC-CCA is not species-specific, the test should be used with caution in co-endemic areas, needing secondary testing using laboratory PCR or urinary filtration with microscopy to confirm results (Lorenz et al., 2025). The POC-CCA test, therefore, appears not to be useful in practice or for prevalence screening for *S. haematobium*. It is, however, a sensitive screening tool in endemic areas for intestinal schistosomiasis caused by *S. mansoni* (sensitivity 93.4%, specificity 63.5%) (Alemu et al., 2025).

The most promising studies use improved reference standards such as PCR, double/triple microscopy, or composite tests. Microscopy is an imperfect reference standard that introduces bias for tests that are more sensitive than microscopy. Five of the nine tests eligible for this review were found to have a high false positive rate, which indicates that the test detects more infections than the reference test. Therefore, specificity results for LAMP, SmCTF-RDT, and ICT-RDT are possibly biased and need to be verified in future accuracy studies that use a higher quality reference test. Future test performance studies in low-prevalence areas should use higher-quality reference tests instead of single microscopy to improve and verify the accuracy of the test performance results of this review.

### Study limitations

A major strength of this review was the robust methodological approach, considering the challenges in diagnosing schistosomiasis. However, meta-analyses require two or more studies with a similar experimental design to enable accurate comparison. In this review, four or less highly heterogeneous studies were available for six of the nine index tests analysed. This resulted in low certainty for most of the tests evaluated in this review. We were able to conduct an adequate meta-analysis for POC-CCA, LAMP, and portable microscope tests, as there were more than four studies for each. Reference test bias occurred in more than 40% of the studies, where single microscopy was used as the reference test in areas with an infection prevalence of <50% among school-aged children, resulting in low specificity results. The specificity results may therefore not be accurate for all nine of the tests in this review.

## Conclusion

Diagnostic gaps have been recognized in areas in Africa where *S. haematobium* is the predominant species of schistosomiasis infection. This review has identified possible tests that have the potential to replace conventional microscopy, particularly in low-resource areas where patients have limited access to health facilities. The rapidity of a test, and its result at the patient’s side is of great value to patients who otherwise may not return readily to receive their results, especially in areas with few or poorly equipped health facilities. We have identified two molecular tests, RPA and LAMP, that can be implemented to diagnose acute, sub-acute, and chronic stages of urogenital schistosomiasis in field/mobile laboratories in poor resource areas with some training of laboratory staff and minimal equipment. We have also identified portable and AI-Assisted microscopes as highly sensitive and rapid screening tests for *S. haematobium* in endemic areas of high prevalence/infection intensity of >50%. Two serological lateral flow screening tests ICT-RDT and SmCTF-RDT are highly sensitive tests able to diagnose all species of *Schistosoma*, and effective for mapping and prevalence screening with rapid results of two hours or less. Both tests have a low specificity result, which may be compromised by reference test bias, and need further testing to confirm their diagnostic accuracy in endemic areas.

An effective control program should implement testing of populations after mass drug administration to determine whether transmission interruption has occurred in a particular area. We have identified RPA as a highly accurate rapid test that functions optimally in low-prevalence settings of up to 2.5% infection prevalence. For the implementation of successful control programs in endemic areas, a test-and-treat strategy may be of great value for improving the support of parents, teachers, and community leaders in gaining the consent of the recommended 75% of a community for mass drug administration strategies. Such programs, however, need to become routine and sustained. Furthermore, identifying and treating patients with chronic or low levels of infection is of paramount value to prevent further transmission of this disease and to prevent the debilitating, long-term morbidities associated with untreated urinary schistosomiasis.

## Data Availability

The original contributions presented in the study are included in the article/supplementary material. Further inquiries can be directed to the corresponding author.
