# Correction: Diagnostic accuracy of rapid and point-of-care tests for *Schistosoma haematobium* in African populations: a systematic review and meta-analysis

**DOI:** 10.3389/fpara.2026.1960349

**Published:** 2026-08-18

**Authors:** Erica Draxl, Eleanor Ochodo, Pavitra Pillay, Ntombizodwa Ndlovu

**Affiliations:** 1 School of Public Health, Faculty of Health Sciences, University of Witwatersrand, Johannesburg, South Africa; 2 Centre for Global Health Research, Kenya Medical Research Institute, Kisumu, Kenya; 3 Centre for Evidence-Based Health Care, Department of Global Health, Faculty of Medicine and Health Sciences, Stellenbosch University, Stellenbosch, South Africa; 4 Department of Biomedical and Clinical Technology, Faculty of Health Sciences, Durban University of Technology, Durban, South Africa

**Keywords:** field-based diagnostic tests, low-resource settings, rapid diagnostic tests, *Schistosoma haematobium*, urogenital schistosomiasis

The funder “Horizon Europe” was erroneously omitted.

The correct **Funding** statement should read:

“The author(s) declared that financial support was received for this work and/or its publication. The research leading to these results has been partially funded by Horizon Europe, Grant Agreement no. 101057853 (DUALSAVE-FGS). The funders had no role in study design, data collection and analysis, decision to publish, or preparation of the manuscript.”

The original version of this article has been updated.

