# Supplementary material for: Diagnostic accuracy of rapid and point-of-care tests for Schistosoma haematobium in African populations: a systematic review and meta-analysis
Source: Front Parasitol. 2026 Jul 22;5:1830789. doi: 10.3389/fpara.2026.1830789 (PMC13439233; doi:10.3389/fpara.2026.1830789)
Supplement: Supplementary file 1 [file SupplementaryFile1.docx]

Supplementary Material for Frontiers Journal Article

**Diagnostic Accuracy of POC and Rapid Tests for Schistosoma haematobium in African populations. A systematic review and meta-analysis: 2026**

**Additional File 1: PRISMA DTA Checklist**

**Additional File 2: Quality Appraisal**

The following criteria were followed when performing the QUADAS-2 Risk assessment:

**Risk of Bias**

Patient selection:

In cross sectional studies: consecutive/random sampling was considered LOW risk

In Case control studies: HIGH risk

In retrospective/current hospital or clinical records: HIGH risk

ANY study using records from patients attending clinics: Bias for patient selection is HIGH

Reference Standard

Single microscopy in low prevalence setting: Bias for Reference test is HIGH

**Applicability concerns:** Applicability judgements depend on precise formulations of review questions. Here also, the “unclear’ category should only be used when insufficient data are reported.

We ensured that the study applies to the review question: PICOT:

P=African populations living in endemic and non-endemic areas, I=tests, eligible rapid and point-of-care tests with a timing of 2 hours for detection for *S. Haematobium* infection C=Laboratory based reference tests, O=Performance accuracy T= Time period January 2000 to July 2025.

Figure 1: Risk of bias and applicability concerns summary: authors' judgements about each domain

**Additional File 3: Individual summary ROC plots for each index test group**

Summary ROC-plots were generated from the data for tests with 4 records or more, producing a summary curve representing how the sensitivity and specificity of the included studies trade off with each other. The summary point, 95% confidence and 95% prediction region can be seen revealing high heterogeneity for POC-CCA and LAMP, and moderate heterogeneity for Portable microscopes.

C: Portable microscope

B: LAMP

A: POC-CCA

Figure 2: HSROC plot for POC-CCA (A), LAMP(B) and Portable microscopes (C) with 95% confidence region revealing high heterogeneity for A and B, respectively and moderate heterogeneity for C.

C: ICT-RDT

B: IHA

A: RPA

Figure 3: HSROC plot for RPA (A), IHA (B) and ICT-RDT (C), black dot represents summary point

**Additional File 4: Exploratory Analysis for individual tests**

### 1. **Exploratory analysis for IHA**

SAC: School aged children.

Figure 4: Forest plot of sensitivity and specificity for IHA rapid diagnostic test including co-variates

Figure 5: Summary ROC plot of IHA for subgroup analysis of prevalence setting

The IHA test is clearly not suited for low prevalence settings such as those done by Yameny (red line) and performs better in a higher prevalence (black line).

2.**Exploratory analysis for ICT-RDT**

Figure 6: Forest plot for ICT-RDT including covariates

A

B

Figure 7: SROC plot for ICT-RDT for subgroup prevalence setting (A), and reference test (B)

3. **Exploratory analysis for POC-CCA**

Visually, the heterogeneity for POC-CCA can be considered high, as indicated by sensitivity values in the forest plot (Figure 8), and the large 95% prediction region in the SROC plot. (Figure 2A). Sampling bias and incorrect reporting occurred in 2 studies. Various prevalence settings may be the reason for heterogeneity. It appears that POC-CCA is not suitable for *S. haematobium* diagnosis in endemic areas, although low prevalence of *S. haematobium* had improved accuracy results for POC-CCA.

SAC= School aged children.

3.1. Prevalence setting and age-group for POC-CCA

Figure 8: Forest plot of sensitivity and specificity of POC-CCA including co-variates

In Figure 9(A), the study by Stothard (black line) was performed in an endemic region with a high prevalence, and yet he did not find that the test could diagnose a positive test at all. The TP rate was 0 in his study. The studies that were performed in a low-prevalence endemic area had a better sensitivity than other settings (green line), for both co-endemic areas and Sh prevalence areas.

The study which included a population of all ages had better diagnostic accuracy results compared to school-aged children for the POC-CCA. Both groups still gave low accuracy results.

B

A

Figure 9: Summary ROC plot of POC-CCA for subgroup analysis of prevalence setting(A) and age group (B)

4. **Exploratory analysis for LAMP**

Results for the LAMP molecular test reveal high heterogeneity. Specificity results are reduced by the study by Gandasegui (2018) (b) in school-aged children, which used an imperfect field- based DNA extraction method, the rapid heat LAMPellet assay, while all other studies had improved DNA extraction methods. The PCR reference standard reveals higher accuracy results than using single microscopy, which may be a reason for the high heterogeneity for LAMP.

Figure 10: Forest plot of sensitivity and specificity of LAMP including co-variates

4.1 Prevalence setting and reference standard for LAMP

B

A

Figure 11: Summary ROC plot for LAMP based on prevalence setting (A) and Reference test (B).

.

4.2 Age group for LAMP

LAMP is suited for populations of all ages, and have reduced sensitivity results for school-aged children

Figure 12 Summary ROC plot for LAMP based on Age group

**5. Exploratory Analysis for RPA**

The forest plot and SROC plots for the 3 studies for RPA test exhibit low heterogeneity between the studies, with both high results for sensitivity and specificity

Figure 13: Forest plot of sensitivity and specificity of RPA including co-variates

5.1. SROC plots for RPA subgroups: Reference tests and Age group

B

A

Figure 14 Summary ROC plot for RPA based on reference standard (A) and age group (B)

**6. Exploratory Analysis of Portable/ mobile-phone Microscope**

The forest plot for the portable/ mobile-phone microscope reveals significant differences in sensitivity and homogeneous results for specificity. Sources of heterogeneity are the device type, as three different devices are analysed. The Cellscope uses the light and camera lens from the mobile phone itself, whereas the Newton NM1 and the UCLA novel microscope use their own light source and lens. The Schistoscope is an AI-assisted microscope that combines the lens of the mobile phone camera with its own device lens. These differences may be a reason for heterogeneity in the accuracy results, where portable microscopes devices have higher accuracy than the Schistoscope and Cellscope subgroups.

Figure 15: Forest plot of sensitivity and specificity for the mobile microscope

6.1 SROC plot for subgroup Portable Microscopy Device Type

Figure 16 Summary ROC plot for Portable Microscope based on device type

6.2 Age Group and Prevalence Setting for Portable Microscope

B

A

Figure 17. Summary ROC plot for Portable microscope based on age-group (A) and prevalence setting (B).
